# A scoping review protocol to map the evidence on the risks and benefits of population based diabetic foot screening

**DOI:** 10.12688/hrbopenres.13585.1

**Published:** 2022-07-11

**Authors:** Jennifer A. Pallin, Caroline McIntosh, Paul Kavanagh, Sean F. Dinneen, Patricia M. Kearney, Claire M. Buckley

**Affiliations:** 1 School of Public Health, University College Cork, Cork City, Cork, T12 XF62, Ireland; 2 Discipline of Podiatric Medicine, National University of Ireland, Galway, Galway City, Galway, H91 TK33, Ireland; 3 Health Intelligence Unit, Strategic Planning and Transformation, Jervis House, Jervis Street, Health Service Executive, Dublin 1, D01 W596, Ireland; 4 Department of Public Health and Epidemiology, Royal College of Surgeons in Ireland, Dublin 2, D02 YN77, Ireland; 5 School of Medicine, National University of Ireland Galway, Galway City, Galway, H91 TK33, Ireland; 6 Centre for Diabetes, Endocrinology and Metabolism, University College Hospital, Galway, Galway, H91 YR71, Ireland

**Keywords:** diabetic foot disease, population health screening, scoping review, principles of screening, diabetic foot ulcer prevention

## Abstract

**Background**: Diabetic foot ulcers are one of the most common lower extremity complications of diabetes, with the lifetime risk of a person developing a DFU estimated to be as high as 34%. It is recommended that those with diabetes receive an annual review of their feet, by a trained healthcare professional, to identify risk factors for ulceration and allow for subsequent risk stratification, patient education and provision of appropriate care to prevent ulceration and amputation. Internationally, while many countries have a diabetic foot care pathway, it is not a structured population health screening programme unlike other areas of preventive care for people with diabetes such as retinopathy screening. A structured diabetic foot screening pathway could allow for earlier identification of the at-risk foot. However, the introduction of any population screening programme should meet the Wilson and Jungner principles of screening. This paper presents a protocol for a scoping review of existing evidence on screening for the at-risk-foot against the Wilson and Junger principles.

**Methods: **The scoping review will be conducted in line with the six-stage methodological framework by Arksey & O’Mally and the Joanna Briggs Institute (JBI) scoping review methodology. Medline (EBSCO), Scopus, ScienceDirect and EMBASE databases will be searched. Studies relating to the burden of diabetic foot ulcers, their pathophysiology and screening tests for peripheral neuropathy and peripheral artery disease, and screening programmes will be included. A data extraction tool will be used to facilitate a chronological narrative synthesis of results.

**Results: **These will be reported in line with the Preferred Reporting Items for Systematic Reviews and Meta-analyses extension for Scoping Reviews (PRISMA-ScR).

**Conclusion: **This scoping review will evaluate and map the evidence surrounding diabetic foot ulcers using the Wilson and Jungner principles of screening as a framework.

## Introduction

Diabetic foot ulcers are one of the most common lower extremity complications of diabetes, with the lifetime risk of a person with diabetes developing a DFU estimated to be as high as 34% ^1^. Diabetic foot ulcers increase the risk of lower extremity amputation and place a significant burden on the individual and the health system ^2– 5^. They also lead to an increased risk of death, which increases further when a person undergoes an amputation ^1, 6, 7^. The average 5-year mortality is 30.5% after a DFU, 46.2% after a minor amputation and 56.6% after a major amputation ^8^. In addition to the increased risk of death, DFUs are a leading cause of the global burden of disability, accounting for 2.1% of global years lived with a disability ^9, 10^. They also negatively impact an individual’s health related quality of life ^11– 13^, lead to increased risk of depression ^14, 15^ and increased financial stress ^16^.

The presence of one or more risk factors (e.g. foot deformity, peripheral neuropathy, peripheral artery disease) places those with diabetes at an increased risk of developing ulcerations and progressing to amputation ^17– 20^. However, these risk factors can be identified through regular assessments by appropriately trained healthcare professionals ^17– 20^. The International Working Group by the Diabetic Foot (IWGDF), an international consortium of clinical and academic experts in diabetic foot disease, recommends that those who have no previously identified risk factors for ulceration should be reviewed annually for signs and symptoms of loss of protective sensation and peripheral arterial disease. The rationale being that, in general, patients who have no risk factors are at a very low risk of developing a DFU, whereas evidence suggests a loss of protective sensation and/or peripheral arterial disease is predictive of DFU development ^21, 22^. They then recommend that those who have loss of protective sensation and/or peripheral arterial disease should be screened for presence of foot deformity, excess callus, pre-ulcerative lesions on the foot and for a history of foot ulceration or lower-extremity amputation ^22^. Table 1 outlines the risk factors, their definitions and associated screening tests, as outlined by the IWGDF.

**Table 1.** Risk factors for Ulceration and Associated Screening Tests.

Risk Factor	IWGDF Definition ^31^	Recommended Screening Test
**Loss of Protective** **Sensation**	The inability to sense light pressure.	• 10g Monofilament • Tuning fork or biothesiometer/ neurothesiometer
**Peripheral Arterial** **Disease**	Obstructive atherosclerotic vascular disease with clinical symptoms, signs, or abnormalities on non-invasive or invasive vascular assessment, resulting in disturbed or impaired circulation in one or more extremities.	• Pulse palpation • Pedal Doppler arterial waveforms
**Deformity**	Alterations or deviations from normal shape or size of the foot, such as hammer toes, mallet toes, claw toes, hallux valgus, prominent metatarsal heads, pes cavus, pes planus, pes equinus, or results of Charcot neuro-osteoarthropathy, trauma, amputations, other foot surgery or other causes.	• Visual inspection for alternations or deviations from normal shape or size of foot
**Callus**	Hyperkeratosis caused by excessive mechanical loading.	• Visual inspection
**Pre-ulcerative signs**	Foot lesion that has a high risk of developing into a foot ulcer, such as intra-cutaneous or subcutaneous haemorrhage, blister, or skin fissure not penetrating into the dermis in a person at risk.	• Visual inspection
**History of ulceration** **(also referred to as** **foot in remission)**	Intact skin and absence of infection of the complete foot after healing of any foot ulcer(s).	• Visual inspection and/or verbal history
**History of foot** **amputation**	Resection of a segment of a limb through a bone or through a joint.	• Visual inspection

As this paper focuses on DFUs and associated risk factors for onset, it is important to note that throughout the literature, the terms examination, assessment, screening and foot check are used to describe the annual review of the foot in diabetes to identify risk factors for ulceration ^23– 27^. The term screening is defined as the “testing of people who either do not have or have not recognized the signs or symptoms of the condition being tested for” and “where the stated or implied purpose is to reduce risk for that individual of future ill health in relation to the condition being tested for, or to give information about risk that is deemed valuable for that individual even though risk cannot be altered” ^28^. In addition, screening tests are not diagnostic, but are used to identify a subset of the population who are at higher risk of a health problem or a condition, who should be referred for additional testing/assessment to determine whether the disease is present. In contrast, definitions around assessment and examination describe the process of investigating or inspecting to make a definitive diagnosis ^29, 30^. The term diabetic foot screening will be used within this protocol to describe the annual review of the foot in diabetes.

In the case of diabetic foot ulcers, screening is the process of identifying risk factors for ulceration so those with risk factors can be referred for further assessment or diagnosis, and if necessary, receive appropriate management or treatment for the risk factor identified by skilled healthcare professionals trained in comprehensive assessment of the lower limb. For example, following further assessment those with peripheral arterial disease may need referral to vascular specialists to restore blood flow to the affected arteries ^32^. The IWGDF also makes recommendations on screening frequency depending on risk factors identified. As outlined in Table 2, the IWGDF recommends that those who have no loss of protective sensation or peripheral arterial disease are stratified as risk level 0 and should be screened annually, whereas those identified as having risk factors are stratified within a higher risk level and should be assessed more frequently and ideally by a foot health specialist (e.g., a podiatrist) ^22^. 

**Table 2.** IWGDF Risk Stratification for the Foot in Diabetes.

Risk Level	Risk of Developing an Ulcer	Characteristics	Frequency of foot screening
0	Very Low	No LOPS * *or* PAD **	Annually
1	Low	LOPS *or* PAD	Every 6–12 months
2	Moderate	LOPS + PAD, *or* LOPS + foot deformity, *or* PAD + foot deformity	Every 3–6 months
3	High	LOPS *or* PAD, and one or more of the following: • history of a foot ulcer • a lower-extremity amputation (minor or major) • end-stage renal disease	Every 1–3 months

*LOPS: Loss of Protective Sensation; **PAD: Peripheral arterial disease

The IWGDF also recommends that annual foot screenings be carried out as part of the patient’s regular diabetes care. Implementation of the IWGDF recommendations into clinical guidelines differs depending on the geographical location ^22, 26, 33, 34^. Some international guidelines recommend that an annual diabetic foot screening be carried out by the person’s primary doctor or nurse whereas others recommended it be carried out by a healthcare professional with no specific advice on when, where or by whom ^26, 34, 35^. However, the diabetic foot care programmes that are present are not situated within a quality assured structured population health screening programme ^23– 25, 36^. Quality assurance processes are necessary to minimise error and improve performance using explicit standards ^28^. In the case of diabetic foot screening, this lack of quality assurance may be contributing factor to why many patients do not receive an annual diabetic foot screening or are only being referred for specialist diabetic foot care when they present with a “foot concern” ^37– 39^. It is also important to note that although diabetic foot screening is recommended as part of a pathway of care, and in some countries as part of structured diabetes care, it can be costly and complex to organise, particularly given the increasing prevalence of diabetes and the limited time allotted for primary care visits ^22^.

A potential alternative to current approaches would be to move diabetic foot screening into a structured quality-assured population health screening programme ^40, 41^. However, there are principles to consider when deciding whether to implement a screening programme for a given disease or health problem ^42, 43^. These principles (See Table 3) were first outlined by Wilson and Jungner (1968) for the WHO and they set the gold standard for determining the appropriateness of screening for a disease ^43^.

**Table 3.** Wilson and Jungner (1968) Principles of Screening.

Category	Criteria
**The Condition**	• The condition should be an important health problem • There should be a recognisable latent or early symptomatic stage.
**The Screening Method**	• There should be a suitable test or examination • The test should be acceptable to the population
**The Treatment**	• There should be an accepted treatment for patients with recognized disease. • There should be an agreed policy on whom to treat as patients. • There should be agreed evidence-based policies covering which individuals should be offered interventions and the appropriate intervention to be offered. • Facilities for diagnosis and treatment should be available
**The Screening Programme**	• The cost of case-finding (including diagnosis and treatment of patients diagnosed) should be economically balanced in relation to possible expenditure on medical care as a whole. • Case-finding should be a continuing process and not a "once and for all" project.

Over the years, these principles have informed decisions internationally on population health screening guidelines ^42, 44, 45^. A recent systematic review and Delphi consensus process identified, synthesised and consolidated all principles of screening published since Wilson and Jungner’s ^42^. While the review assessed that many aspects of Wilson and Jungner’s criteria are still relevant today, it identified a shift towards principles that focus on operational or implementation issues of whole screening programmes, rather than just screening for the disease or condition in question. These operational or implementation issues including screening programme acceptability and ethics, benefits and harm, and quality and performance management are not addressed by Wilson and Jungner ^42^. This scoping review will evaluate the evidence on population screening for the at-risk foot in diabetes using both the Wilson and Jungner, and the more recent Dowbrow and colleagues, principles of screening to ensure operational and implementation issues relating to diabetic foot are captured within the scoping review.

## Methods

A scoping review is defined as “a form of knowledge synthesis that addresses an exploratory research question aimed at mapping key concepts, types of evidence, and gaps in research related to a defined area or field by systematically searching, selecting, and synthesizing existing knowledge” ^46^. The scoping review outlined within this protocol will be conducted in line with the framework by Arksey and O’Malley (2005) and the Joanna Briggs Institute (JBI) scoping review methodology ^47, 48^. In line with recommendations by Arksey and O’Malley (2005), the authors recognise that the scoping review process may not be linear, and the researchers may need to engage with each stage in a reflexive way and potentially repeat steps to ensure the literature is covered comprehensively ^48^. Where changes need to be made, these will be outlined in the final scoping review report, which will be reported in accordance with the Preferred Reporting Items for Systematic Reviews and Meta-analyses (PRISMA) extension for Scoping Reviews (PRISMA-ScR) ^49^.

### Step 1: Identifying the research question

The research questions within this scoping review protocol are being guided by the Wilson and Jungner, and Dobrow and colleagues, principles of screening ^42, 43^. Therefore, the overarching aim of this scoping review is to evaluate and map the evidence surrounding diabetic foot ulcers, and screening for risk factors for ulceration, using the principles outlined by Wilson and Jungner. In line with recommendations by Dobrow and colleagues ^42^, the scoping review will also evaluate the evidence on the clinical, social, and ethical acceptability of a diabetic foot screening programme to participants, health professionals and society. The research questions include: 

1. Are diabetic foot ulcers an important health problem? (i.e., increasing prevalence or incidence, or causing substantial morbidity and/or mortality). 

2. Is the natural history of diabetic foot ulcers clearly understood?

3. Is the screening test for risk factors for diabetic foot ulcers safe, simple, reliable, validated and acceptable to the population? 

4. Are there effective interventions for those identified as at-risk, with evidence that intervention at a pre-symptomatic phase leads to better outcomes for the screened individual compared with usual care? 

5. Is there evidence that diabetic foot screening is cost-effective? 

6. Is there evidence that a diabetic foot screening programme would be clinically, socially and ethically acceptable to screening participants, health professionals and society? 

### Step 2: Identifying relevant studies

***Eligibility criteria:*** Only those published in English will be included. Systematic reviews, meta-analysis, case-control, case-series, randomised control trial, qualitative and economic evaluation study designs will be included. As outlined in Table 4, inclusion and exclusion criteria have been informed by the Wilson and Jungner principles of screening ^42, 43^. Although deformity, skin and nail pathologies and/or history of ulceration have been identified as risk factors for ulceration, this review will only include screening tests that screen for loss of protective sensation and peripheral arterial disease. The rationale being that the IWGDF recommends that screening for deformity, nail and skin pathologies and history of ulceration and/or amputation be carried out only when loss of protective sensation and/or peripheral arterial disease are present. In addition, evidence suggests that loss of protective sensation and/or peripheral arterial disease are the most significant predictors of foot ulceration ^21, 22^

**Table 4.** Eligibility Criteria.

Category	Inclusion Criteria	Exclusion Criteria
**The Condition**	• Publications that report on the: ◦ epidemiology of diabetic foot ulcers. ◦ associated mortality and/or morbidity of diabetic foot ulcers. ◦ cost to the health service of diabetic foot ulcers ◦ natural history or pathophysiology of diabetic foot ulcers.	• Any publication relating to other diabetic related lower extremity complications (e.g. amputations, Charcot Neuroarthropathy, diabetic foot infections) or leg ulcers.
**The Screening** **Method**	• Studies that report on whether the screening tests * for the foot in diabetes are simple, safe, precise, reliable and validated. • Screening tests recommended by the IWGDF will be included. For LOPS these include: ◦ 10-g Semmes Weinstein monofilament ◦ 128 Hz Tuning fork ◦ Biothesiometer ◦ Neurothesiometer For PAD these include: ◦ Palpation of pulses ◦ Pedal Doppler arterial waveforms	• Studies that report on the use of these screening tests in those who do not have a diagnosis of diabetes. • Studies that report on the use of these screening tests for secondary prevention of diabetic foot ulcers.
**The** **Treatment**	• Studies that report on treatment and management pathways of those at-risk of developing a diabetic foot ulcer • Studies that report on the treatment and/or management of the foot in diabetes with one or more of the following risk factors: ◦ Loss of protective sensation ◦ Peripheral arterial disease	• Publications relating to the treatment and management (e.g. wound dressings, offloading techniques) of diabetic foot ulcers will be excluded. • Studies that report on secondary prevention of diabetic foot ulcers.
**The Screening** **Programme**	• Studies that report on: ◦ diabetic foot screening and its effectiveness, and cost-effectives, in preventing diabetic foot ulcers. ◦ the clinical, social, and ethical acceptability of diabetic foot screening to participants, health professionals and society. ◦ pathways of care that include an annual diabetic foot screening, assessment or examination	

*At-Risk Diabetic Foot: A foot in a person with diabetes that is at-risk of developing a diabetic foot ulcer as they have peripheral neuropathy and/or peripheral vascular disease ^22^.

***Search strategy*.** In line with JBI recommendations, a three-step process for applying a search strategy will be implemented ^50, 51^. ****Step one**** has already been carried out in consultation with a research librarian and involved a broad search on Medline (EBSCO) to identify key search terms (See Table 5 for key search terms). These terms are associated with the concepts of diabetic foot ulcers and diabetic foot screening. To ensure all relevant papers are identified, the terms examination, assessment, and screening will be used during the search. Boolean operators AND, OR and proximity operators will combine search terms to ensure the search strategy is as efficient as possible and to reduce the risk of capturing irrelevant material. 

***Step two*** will involve using the identified key words outlined in Table 5 on the Scopus, ScienceDirect and EMBASE databases. Key words and search terms will be adapted to suit the relevant databases. ***Step three*** will involve a search of the reference lists of the selected studies to identify any additional relevant studies. In addition, a web search will be conducted using “Google” and “Google Scholar”.

**Table 5.** Medline (EBSCO) Search Strategy.

Category 1: The condition
**Core Search:** (MH "Diabetic Foot") OR “diabetic foot ulcer" OR "diabetic foot disease OR “diabetic foot syndrome” **Combined with each of the following using Boolean operator term AND** 1. (MH "Incidence") OR (MH "Prevalence") OR (MH "Epidemiology") OR (MH "Epidemiologic Studies") OR (MH "Epidemiologic Factors") 2. (MH "Mortality") OR (MH "Morbidity") OR (MH "Life Expectancy") 3. (MH "Quality of Life") 4. (MH "Risk Factors") OR (MH "Causality") 5. (MH "Health Care Costs") OR (MH "Health Care Sector") OR (MH "Economics, Hospital") OR (MH "Health Expenditures") OR (MH "Cost of Illness")
Category 2: The Screening Test
**Screening tests for Peripheral Neuropathy** **Core Search:** (MH "Diabetic Neuropathies”) OR (MH "Diabetic Foot") **Combined with each of the following using Boolean operator term AND** 1. “monofilament” OR “neurothesiometer OR “biothesiometer” OR “tuning fork” OR “vibratip” 2. (MH "Early Diagnosis") 3. (MH "Diagnostic Tests, Routine") OR (MH "Point-of-Care Testing") ) 4. (MH "Risk Assessment") OR (MH "Diagnostic Tests, Routine") OR (MH "Physical Examination") **Screening Tests for Peripheral Vascular Disease** **Core Search:** (MH"Peripheral Vascular Diseases") OR (MH "Peripheral Arterial Disease") OR (MH "Vascular Diseases) OR (MH "Microcirculation")) OR (MH "Diabetic Foot") **Combined with each of the following using Boolean operator term AND** 1. (MH "Pulse Wave Analysis") OR (MH "Pulse") OR (MH "Ultrasonic Waves") OR (MH Ultrasonography, Doppler, Pulsed") OR (MH "Traditional Pulse Diagnosis") 2. “doppler” 3. “palpation” 4. (MH "Early Diagnosis") 5. (MH "Diagnostic Tests, Routine") OR (MH "Point-of-Care Testing") 6. ((MH "Risk Assessment") OR (MH "Diagnostic Tests, Routine") OR (MH "Physical Examination"))
Category 3: The Treatment
**Core Search:** (MH "Diabetic Foot") OR (MH "Diabetic Neuropathies") OR (MH "Diabetic Angiopathies") **Combined with each of the following using Boolean operator AND** 1. (MH "Population Surveillance") OR (MH "Public Health Surveillance") OR "surveillance" 2. (MH "Disease Management") 3. (MH "Therapeutics") 4. (MH "Standard of Care")) 5. (MH "Critical Pathways") 6. (MH "Patient Care Team") 7. ("treatment" OR "management") 8. "risk stratification"
Category 4: The Screening Programme
**Core Search:** ((MH "Diabetic Foot") OR (MH "Diabetes Complications")) **Combined with Boolean operator term AND** 1. ((MH "Physical Examination") OR (MH "Risk Assessment") OR (MH "Symptom Assessment") OR (MH "Mass Screening") OR (MH "Early Diagnosis")) 2. ((MH "Ethics") OR (MH "Patient Acceptance of Health Care")) ((MH "Costs and Cost Analysis") OR (MH "Health Expenditures") OR (MH "Cost Savings") OR (MH "Cost-Benefit Analysis")) 3. (MH "Primary Prevention") OR (MH "Preventive Medicine") OR (MH "Secondary Prevention") OR (MH "Preventive Health Services") OR (MH "Diagnostic Screening Programs") OR (MH "Early Diagnosis") 4. "prevention and control" 5. (MH "Diagnostic Screening Programs") OR "program" 6. (MH "Patient Care Team")

### Step 3: Study selection

***Search selection and screening*.** Once a search has been conducted within a database, it will be documented (date, search terms, results per string) in an excel document. Studies found will be imported into the bibliographic reference manager, Mendeley, and any duplicates removed. The online tool COVIDENCE ( www.covidence.org) will be used for screening against eligibility criteria. Screening and selection will be carried out in two steps. During step 1, the lead reviewer will screen all titles and abstracts and a second reviewer will screen 50% of titles and abstracts. A second reviewer will be involved to ensure all relevant studies are selected for review, and to reduce the risk of selection bias ^52, 53^. Where consensus cannot be met, a third reviewer will be consulted. Once suitable papers have been selected, full text screening will be carried out by the lead reviewer on all articles that meet the inclusion criteria. The second reviewer will screen 25% of the articles. Where consensus cannot be met, a third reviewer will review the article and decide whether it should be included in the review. Interrater reliability, using the kappa score, will be calculated to evaluate the extent of agreement between reviewers ^54^. A PRISMA flow diagram will be created to ensure transparency of reporting ^49^.

### Step 4: Charting the data

***Data extraction*.** Once articles have been identified for inclusion within the review, data relating to diabetic foot disease, screening tests for peripheral neuropathy and peripheral vascular disease, treatments and diabetic foot screening programmes will be extracted and summarised in an excel table. In line with recommendations from JBI, this form will be piloted on ten full text articles by two of the reviewers to ensure consistency ^55^. Any discrepancies that arise will be discussed by the full research team. However, given the iterative nature of scoping reviews, the authors expect that the data extraction form may need to be adjusted. Critical appraisal will be carried out using the Joanna Briggs Institute critical appraisal tools ^56^. These tools will used as they allow for critical appraisal of all study designs to be included in this scoping review.

### Step 5: Collating, summarising and reporting the results

***Data analysis and summary*.** A narrative synthesis approach will be employed to answer the review questions. Narrative synthesis is an approach where the authors carry out a systematic review and synthesis of findings from multiple studies and rely primarily on the use of words and text to summarise and explain the findings. It also uses a textual approach to ‘tell the story’ of the findings from included studies ^57^. Data will be collected into four standalone categories: 1) The Condition, 2) The Screening Method, 3) The Treatment and 4) The Screening Programme.

### Step 6: Consultation with relevant stakeholders

Following recommendations by Daudt, van Mossei and Scott (2013) to embed consultation throughout the scoping review process, suitable professional stakeholders were invited to be a part of the research team to help guide the research design and questions ^58^. In addition, once findings have been collated, they will be presented to a wider group of key professional stakeholders. These will be recruited using a purposive sampling approach to ensure the researchers identify and select stakeholders who have a particular knowledge or understanding about the research question and population health screening ^59^. These may include policy makers, members of national statutory and/or voluntary bodies, healthcare professionals and researchers.

Furthermore, patient and public representatives of those affected by diabetes will be consulted during the analyses and reporting phases of the scoping review. This patient and public involvement (PPI) panel will ensure that the research conducted, and findings generated and reported are relevant to those affected by diabetes ^60^. During the writing of this protocol, it became evident that different terms (i.e., assessment, examination, or screening) are used throughout the literature to describe the process of annually inspecting the foot in diabetes to identify risk factors for ulceration. The concept of terminology will be discussed with the PPI panel to explore their understandings and preference for terminology used. Any patient and public involvement will be reported in the final scoping review publication using the Guidance for Reporting Involvement of Patients and the Public (GRIPP) short form ^61^.

### Dissemination and ethics

The primary author will deliver results via oral or poster presentations at national and international conferences and publish the scoping review findings in a peer-reviewed journal for wider communication of the results. The authors aim to ensure that data generated and analysed during the scoping review will be included in the published scoping review article; including search results, list of included studies, data extraction spreadsheets and final results, to ensure transparency and reproducibility of the review ^50^. Finally, results from the scoping review will inform further research in the area of diabetic foot screening as part of the lead author’s PhD. As the scoping review outlined within this protocol will use publicly available literature, ethical approval will not be needed.

## Discussion

International guidelines recommend that all patients with diabetes should be assessed at least annually for the presence of the at-risk foot so interventions can be put in place to prevent progression to ulceration and amputation. While some countries have a form of diabetic foot screening, the screening is not part of a structured population health screening programme ^23– 25, 36^. One potential way of improving diabetic foot care and ensuring all patients with diabetes are offered an annual diabetic foot screening is by introducing it into a structured population health screening programme. However, little work has been carried out to examine the evidence on whether introduction of diabetic foot disease into a population health screening programme is an appropriate course of action to prevent diabetic foot related complications ^42, 43^. This review will evaluate and map the evidence surrounding diabetic foot ulcers using the principles outlined by Wilson and Jungner. Findings generated have the potential to evaluate whether introduction of diabetic foot screening into a population health screening programme is appropriate and whether certain areas warrant further research.

## Data availability

No data are associated with this article.
