# Supplementary material for: A scoping review protocol to map the evidence on the risks and benefits of population based diabetic foot screening
Source: HRB Open Res. 2022 Jul 11;5:50. [Version 1] doi: 10.12688/hrbopenres.13585.1 (PMC10432895; doi:10.12688/hrbopenres.13585.1)
Supplement: Peer review history

# Peer review: Reviewer response for version 1

**DOI:** 10.21956/hrbopenres.14839.r34190

**Published:** 2023-08-16

**Authors:** Zhiwen Joseph Lo

**Affiliations:** 1 Nanyang Technological University, Singapore, Singapore

Thank you for the privilege to review. Congrats to the authors for a well-written manuscript. 

 Clearly written methodology for the proposed scoping review. Looking forward to the eventual results and publication. 

 Objectives are clearly described. Appropriate study design. Sufficient details to methodology. Clear presentation of various tables. 

 Reviewed and suitable for indexing.

Is the study design appropriate for the research question?

Yes

Is the rationale for, and objectives of, the study clearly described?

Yes

Are sufficient details of the methods provided to allow replication by others?

Yes

Are the datasets clearly presented in a useable and accessible format?

Yes

Reviewer Expertise:

Diabetic limb salvage

I confirm that I have read this submission and believe that I have an appropriate level of expertise to confirm that it is of an acceptable scientific standard.

# Peer review: Reviewer response for version 1

**DOI:** 10.21956/hrbopenres.14839.r34194

**Published:** 2023-08-16

**Authors:** Cynthia Formosa

**Affiliations:** 1 University of Malta, Msida, Malta; 2 Staffordshire University, Stoke-on-Trent, England, UK

This is a well written article on diabetes foot screening which warrants publishing.

 The arguments made in the paper are valid and actual. The layout of the review is clear. All dissemination and ethical issues are clearly highlighted in the article. The article is well referenced. Framework chosen is appropriate.

Is the study design appropriate for the research question?

Yes

Is the rationale for, and objectives of, the study clearly described?

Yes

Are sufficient details of the methods provided to allow replication by others?

Yes

Are the datasets clearly presented in a useable and accessible format?

Yes

Reviewer Expertise:

diabetes, diabetic foot, diabetes foot screening guidelines

I confirm that I have read this submission and believe that I have an appropriate level of expertise to confirm that it is of an acceptable scientific standard.
